# Maternal regulation of inflammatory cues is required for induction of preterm birth

**DOI:** 10.1172/jci.insight.138812

**Published:** 2020-11-19

**Authors:** Monica Cappelletti, Jessica R. Doll, Traci E. Stankiewicz, Matthew J. Lawson, Vivien Sauer, Bingqiang Wen, Vladimir V. Kalinichenko, Xiaofei Sun, Tamara Tilburgs, Senad Divanovic

**Affiliations:** 1 Department of Pediatrics, University of Cincinnati College of Medicine, Cincinnati, Ohio, USA.; 2 Division of Immunobiology, Cincinnati Children’s Hospital Medical Center, Cincinnati, Ohio, USA.; 3 Medical Scientist Training Program, University of Cincinnati College of Medicine, Cincinnati, Ohio, USA.; 4 Center for Lung Regenerative Medicine,; 5 Division of Reproductive Sciences, and; 6 Center for Inflammation and Tolerance, Cincinnati Children’s Hospital Medical Center, Cincinnati, Ohio, USA.

**Keywords:** Inflammation, Cellular immune response, Cytokines, Mouse models

## Abstract

Infection-driven inflammation in pregnancy is a major cause of spontaneous preterm birth (PTB). Both systemic infection and bacterial ascension through the vagina/cervix to the amniotic cavity are strongly associated with PTB. However, the contribution of maternal or fetal inflammatory responses in the context of systemic or localized models of infection-driven PTB is not well defined. Here, using intraperitoneal or intraamniotic LPS challenge, we examined the necessity and sufficiency of maternal and fetal Toll-like receptor (TLR) 4 signaling in induction of inflammatory vigor and PTB. Both systemic and local LPS challenge promoted induction of inflammatory pathways in uteroplacental tissues and induced PTB. Restriction of TLR4 expression to the maternal compartment was sufficient for induction of LPS-driven PTB in either systemic or intraamniotic challenge models. In contrast, restriction of TLR4 expression to the fetal compartment failed to induce LPS-driven PTB. Vav1-Cre–mediated genetic deletion of TLR4 suggested a critical role for maternal immune cells in inflammation-driven PTB. Further, passive transfer of WT in vitro–derived macrophages and dendritic cells to TLR4-null gravid females was sufficient to induce an inflammatory response and drive PTB. Cumulatively, these findings highlight the critical role for maternal regulation of inflammatory cues in induction of inflammation-driven parturition.

## Abstract 2

Maternal regulation of inflammatory cues is necessary and sufficient for LPS-induced preterm birth in mice.

## Introduction

Preterm birth (PTB), the leading cause of neonatal morbidity and mortality, remains a major public health problem (1, 2). PTB affects approximately 12% of pregnancies worldwide (3). In the United States the rate of PTB has continuously increased from 2014 to 2018 (4, 5). Infection and infection-driven activation of inflammatory responses are thought to be a major cause of “spontaneous” PTB (1, 2, 6). Infection can occur systemically (e.g., listeriosis, influenza, sepsis) or be localized to the intrauterine or intraamniotic compartments (e.g., ascending bacterial infection) (7, 8). Colonization with an infectious agent has been detected in 25%–40% of all preterm deliveries (6). Hence, infection and infection-associated inflammation can regulate the timing of parturition (6). Despite the significant impact of PTB on human health and recognition of infection as a predisposing factor, the cellular and molecular mechanisms underlying inflammation-driven PTB remain undefined (9).

Pathogens can gain access to the intrauterine compartment by ascending the reproductive tract or by hematogenous dissemination through the placenta (6, 10–13). However, the contribution of maternal and fetal responsiveness necessary or sufficient for pathogen-driven cytokine production and induction of PTB is incompletely defined. Different routes of challenge with Toll-like receptor (TLR) ligands, including intraperitoneal (i.p.), intrauterine (i.u.), or intraamniotic (i.a.), are commonly used in reductive studies to define causes of inflammation-driven PTB. LPS-driven activation of TLR4 signaling is a frequently employed experimental mimic of Gram-negative bacterial infection that directly induces PTB in rodent models (14–19). When high doses of LPS are administered i.p., a small proportion of LPS can cross the placental barrier and upregulate prostaglandin release in amniotic fluid (20). However, the route of inflammatory ligand administration, even in the context of the same ligand, can result in vastly different inflammatory responses and pregnancy outcomes (21–23). The ability of different routes of inflammatory challenge to induce PTB is well established. Nevertheless, a direct comparison of the local inflammatory response within the uteroplacental tissues and maternal systemic response dependent on challenge route has not been examined.

TLRs are a family of innate immune receptors that signal the presence of conserved microbial structures, leading to activation of both innate and adaptive immune responses (24). Diverse immune cells (e.g., macrophages, dendritic cells) (25) and nonimmune cells (e.g., epithelial cells, endothelial cells) are known to express TLRs (26). TLRs are highly expressed at the materno-fetal interface by multiple cell types, including trophoblasts, immune cells in the uterine wall, and endothelial cells in the decidual bed (27–29). TLR expression in macrophages and natural killer cells has been correlated with PTB (28, 30–32). Similarly, genetic deletion of TLR4 in mice or antagonism of TLR4 signaling with the small molecule inhibitor (+)-naloxone has been shown to protect from PTB (33, 34).

Even in the experimentally tractable model of TLR-driven PTB, fundamental issues remain unknown. Among these, the necessary anatomical locus of TLR expression (maternal or fetal) required for the deleterious inflammatory origin and the key TLR-expressing cell type (e.g., immune cells, trophoblasts) central for induction of PTB have not been determined. Further, whether inflammatory mediators that contribute to PTB are conserved between systemic and local challenge remains unclear. Here, we examined the contribution of the maternal and fetal compartment in TLR4-driven immune activation and induction of PTB. Our findings suggest that systemic and intraamniotic LPS challenge upregulate similar inflammatory mediators and induce PTB, which is dependent on maternal inflammatory mediator sensing. Although fetal cells were capable of producing IL-6 following LPS stimulation, fetal expression of TLR4 was neither necessary nor sufficient to induce PTB in response to LPS. TLR4 expression by immune cells of myeloid origin was sufficient for driving inflammation-induced PTB, with both macrophages and dendritic cells contributing to this response.

## Results

### LPS purity and route of challenge determine the dose needed to induce PTB.

**Figure 1:**
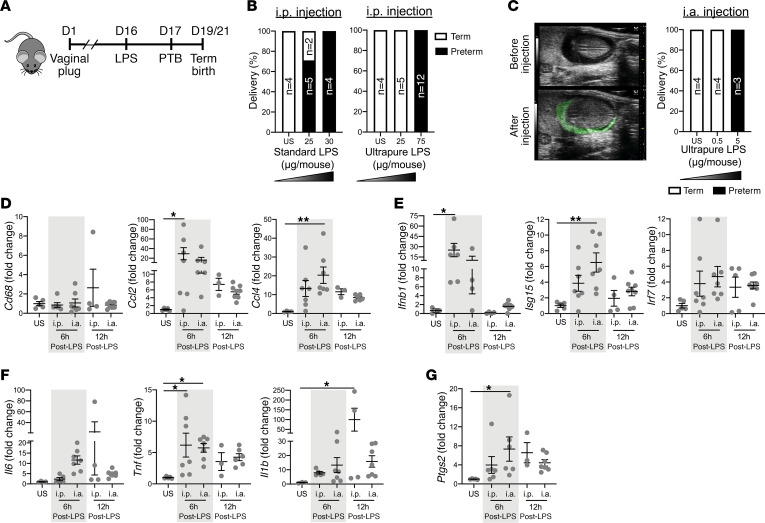
Preterm birth is induced by both systemic and local route of challenge. (**A**) A schematic overview of the approach used to study PTB in gravid mice following LPS challenge. (**B**) Gravid WT mice (*n* = 4–12/condition) were injected i.p. with saline (US, unstimulated) or LPS (standard or ultrapure) at the indicated doses on day 16 of gestation, and the incidence of PTB was quantified. χ^2^ (2 × 3 matrix): standard LPS *P* = 0.0108; ultrapure LPS *P* < 0.0001. (**C**) Ultrasound image taken of an individual amniotic sac on day 16 of gestation. Contrast (green) was included in the i.a. injection, and all the injected fluid was retained within the amniotic sac. Saline or the concentration of ultrapure LPS used in challenge is provided below each bar and was administered as 2 doses in separate amniotic sacs for each uterine horn. Instance of PTB was quantified (*n* = 3–4/condition). χ^2^ (2 × 3 matrix) *P* = 0.0041. (**D**–**G**) Gravid WT mice (*n* = 3–8/condition) were challenged with ultrapure LPS by i.p. (75 μg) or i.a. (5 μg) injection, and mRNA expression in the decidua/myometrium was quantified at 6 and 12 hours postchallenge. Data represent fold change over nonstimulated ± SEM. (**D**) *Cd68*, *Ccl2*, and *Ccl4* mRNA expression. (**E**) *Ifnb*, *Isg15*, and *Irf7* mRNA expression. (**F**) *Il6*, *Tnf*, and *Il1b* mRNA expression. (**G**) *Ptgs2* mRNA expression. (**D**–**G**) ANOVA followed by Tukey’s correction. *P < 0.05, **P < 0.01.

Phosphorylation, fatty acid acylation patterns, and lipid A structure are important contributors to variable vigor of LPS-driven inflammation (35, 36). Interpretation of reduction studies focused on better understanding of LPS-driven immune activation has been complicated by the use of variable and impure reagents (e.g., “LPS” containing other TLR ligands capable of inducing an immune response in the absence of TLR4 or endogenous ligands that are contaminated by LPS) (37). To address the potential for variation because of reagent purity, we compared the ability of standard *E*. *coli*–derived LPS versus ultrapure *E*. *coli*–derived LPS to drive inflammation. HEK293 cells that express TLR2 (termed H2.14), but not TLR4, were stimulated with standard or ultrapure LPS (and Pam2Cys as a positive control). Despite an identical bacterial serotype and concentration, only standard LPS, but not ultrapure LPS, robustly induced IL-8 production in H2.14 cells (Supplemental Figure 1; supplemental material available online with this article; https://doi.org/10.1172/jci.insight.138812DS1). These findings suggested that LPS purity and the quality and strength of the subsequent inflammatory response might impact induction of PTB in vivo. To examine the impact of reagent purity on LPS-driven PTB in our mouse model (Figure 1A), we performed i.p. challenge of pregnant mice with 25 μg standard LPS–induced PTB in 5/7 mice within 24 hours. When the challenge concentration was increased to 30 μg, standard LPS induced PTB in 100% of cases within 24 hours (Figure 1B). In contrast, i.p. challenge of pregnant mice with 25 μg ultrapure LPS (*E*. *coli*) failed to induce PTB, with a 3-fold higher dose (75 μg) required to induce PTB in 100% of the pregnant mice (Figure 1B). Thus the purity of LPS used influenced the dose required to activate pathways resulting in PTB. To avoid potential impact of impurities in the LPS and to rigorously define the relevance of maternal and fetal compartments of TLR4 activation in inflammation-induced PTB, our subsequent studies all used ultrapure LPS.

Systemic inflammatory challenge (i.p. ultrapure LPS administration) was compared with inflammatory challenge originating locally in the fetal compartment (i.a. ultrapure LPS administration). Ultrasound visualization of the amniotic sac allowed for specific targeting of the amniotic fluid. Two amniotic sacs were injected per pregnancy, 1 injection in each uterine horn. Injected fluid was retained within the amniotic sac, as visualized by the addition of contrast (Figure 1C, left panel). Ultrasound-guided i.a., compared with i.p., LPS challenge required a 15-fold lower dose (5 μg vs. 75 μg) to induce 100% PTB (Figure 1C, right panel). Induction of proinflammatory pathways is associated with initiation of labor (38). Because macrophage migration into the uterine lining is detected during labor and macrophages contribute to inflammation-induced PTB (32, 39), markers of macrophage infiltration and activation and uterine tissue inflammation, in the context of i.p. and i.a. LPS-driven PTB, were quantified by reverse transcription quantitative PCR at 6 and 12 hours postchallenge. LPS challenge, by both routes, did not correlate with increased expression of *Cd68* (40) in the decidua and myometrium. However, mRNA expression of chemokines known to promote macrophage tissue infiltration (e.g., *Ccl2* and *Ccl4*) was increased in both i.p. and i.a. injected mice (Figure 1D). Consistent with this finding, at 6 hours postchallenge, placentas (*n* = 9/condition) from both i.a. and i.p. LPS-challenged mice did not shown signs of overt immune cell infiltration compared with unstimulated controls (Supplemental Figure 2). These data suggest that proinflammatory cytokine production, rather than overt immune cell infiltration, contributes to induction of labor in our acute inflammatory model (41). We next examined the induction of inflammatory mediators known to contribute to macrophage activation and induction of PTB at 6 and 12 hours post–LPS challenge (32, 40, 42, 43). LPS challenge resulted in increased expression of type I interferons and interferon-stimulated genes (Figure 1E), immune mediators associated with PTB (43). Similarly, genes encoding proinflammatory cytokines known to promote PTB (e.g., *Il6*, *Tnf*, and *Il1b*) (43–48) were also upregulated at both 6 and 12 hours post–LPS challenge (Figure 1F).

The processes of cervical ripening and dilation, uterine contractions, and rupture of fetal membranes are required for fetal delivery (49). IL-6 and TNF are robust inducers of *Ptgs2* (Cox-2) (50), a well-established mediator of uterine contractility, cervical ripening, and induction of labor (21, 51–53). In line with the expression of inflammatory mediators, *Ptgs2* (Cox-2) expression was increased following LPS challenge (Figure 1G). Together, these data demonstrate that both i.p. and i.a. LPS challenge drove reproductive tissue inflammation and the initiation of PTB. Further, our data show that sensitivity to LPS dose correlated with the increased expression of the above listed parturition-associated mediators.

### Maternal TLR4 expression is sufficient to induce preterm birth.

**Figure 2:**
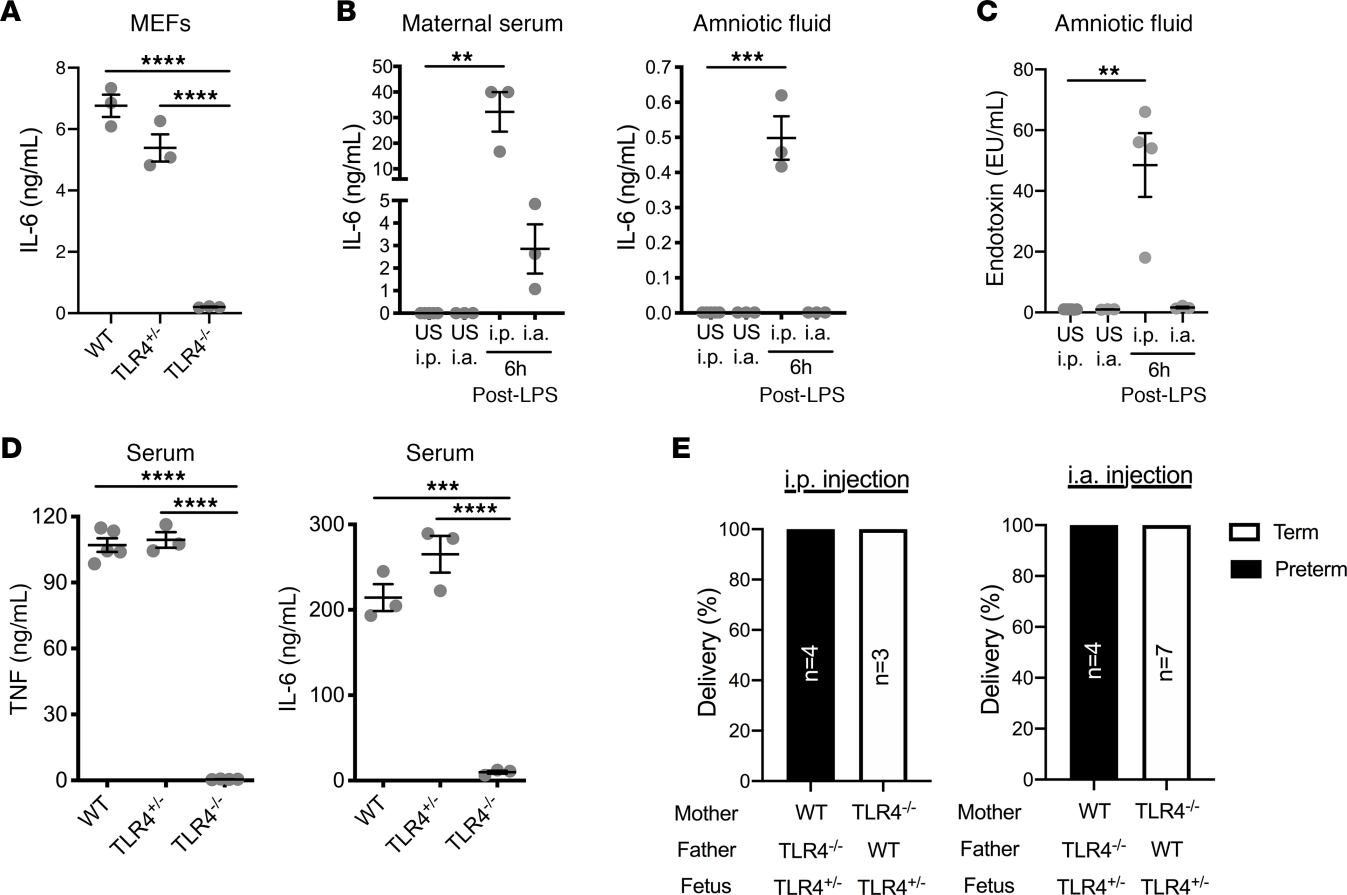
Maternal TLR4 expression is required to induce preterm birth. (**A**) MEFs (*n* = 3/condition) were isolated on day 13 of pregnancy, and the IL-6 response to ultrapure LPS was measured in WT, TLR4-heterozygous, and TLR4-knockout cells. Data represent average ± SEM. (**B**) IL-6 levels in the serum and amniotic fluid of WT mice 6 hours after i.p. or i.a. administration of LPS (75 μg or 5 μg, respectively) or saline on day 16 of pregnancy (*n* = 3/condition). Cytokines were measured in pooled amniotic fluid for each uterine horn and graphed as the average amount per pregnancy. Data represent average ± SEM. (**C**) Endotoxin levels in amniotic fluid of WT mice 6 hours after i.p. or i.a. administration of LPS (75 μg or 5 μg, respectively) or saline on day 16 of pregnancy (*n* = 3–4/condition). Amniotic fluid was pooled for each uterine horn and graphed as the average amount per pregnancy. Data represent average ± SEM. (**D**) Levels of serum IL-6 and TNF determined by in vivo cytokine capture assay (IVCCA) in adult WT, TLR4-heterozygous, and TLR4-knockout mice following i.p. injection with ultrapure LPS (*n* = 3–5/condition). Data represent average ± SEM. (**E**) Gravid WT and TLR4-knockout mice (*n* = 3–7/condition) carrying heterozygous pups were treated at day 16 of pregnancy with i.p. or i.a. ultrapure LPS and instance of PTB was quantified. χ^2^ (2 × 4 matrix) *P* = 0.0004. (**A**–**D**) ANOVA followed by Tukey’s correction. **P < 0.01, ***P < 0.001, ****P < 0.0001.

TLRs are robustly expressed at the materno-fetal interface (32), and when high doses of LPS are administered i.p., a small proportion of LPS is able to access the fetal compartment (20). The fetus can respond to inflammatory challenge and contributes to induction of PTB when exposed to inflammatory stimuli (54). However, the contribution and sufficiency of maternal and fetal TLR4 signaling to drive the inflammatory response necessary to induce PTB have not been defined. The ability of fetal cells to respond to LPS was directly tested via usage of mouse embryonic fibroblasts (MEFs). LPS stimulation was sufficient to induce IL-6 production by both WT and TLR4-heterozygous (TLR4^+/–^) MEF cultures. As expected, such response was completely abrogated in cultured MEFs that lacked TLR4 expression (TLR4^–/–^) (Figure 2A). This finding demonstrated that fetal compartment–specific TLR4, even in the context of heterozygous expression, had the potential to detect and respond to inflammatory stimuli.

To directly assess fetal and maternal cytokine production in response to LPS, IL-6 was measured in the serum and amniotic fluid from pregnant mice at 6 hours after LPS injection. Cytokine levels were overall higher in the i.p. injected compared with the i.a. injected group (Figure 2B). A high IL-6 response was detected in maternal serum as a result of both routes of challenge. However, while low levels of IL-6 were detected in the amniotic fluid of i.p. injected pregnant mice, the IL-6 levels were below the limit of detection (<32 pg/mL) in the amniotic fluid of i.a. injected pregnant mice (Figure 2B). This was consistent with the level of endotoxin measured in amniotic fluid (Figure 2C). These data invoke the possibility that the maternal inflammatory response, at the time point examined, compared with fetal inflammatory response, may have a larger contribution to induction of LPS-driven PTB (55, 56).

To begin to define the contribution of maternal and fetal TLR4 expression in responding to LPS challenge and driving PTB, we sought to specifically restrict TLR4 either to the maternal or to the fetal compartment. Because TLR4 heterozygosity in MEFs was sufficient to allow robust LPS-driven proinflammatory cytokine production (Figure 2A), such sufficiency was tested in vivo. TLR4-heterozygous mice produced similar serum levels of IL-6 and TNF as WT littermates, and cytokine production was not detected in TLR4^–/–^ mice in response to i.p. LPS challenge (Figure 2D). Therefore, TLR4 heterozygosity was sufficient to respond to LPS challenge. To examine the role of fetal expression of TLR4 in LPS-driven induction of PTB, TLR4^–/–^ females were bred with WT males to yield TLR4 expression specifically in the fetal compartment. TLR4^–/–^ mothers carrying TLR4-heterozygous fetuses were challenged i.p. or i.a. with LPS. These pregnancies were fully protected from either i.p. or i.a. LPS-driven PTB (Figure 2E). In contrast, WT or TLR4-heterozygous mothers carrying TLR4-heterozygous progeny were susceptible to LPS-driven PTB (Figure 2E). Together, these findings suggest that maternal expression of TLR4 may play a dominant role in LPS-driven induction of PTB.

**Figure 3:**
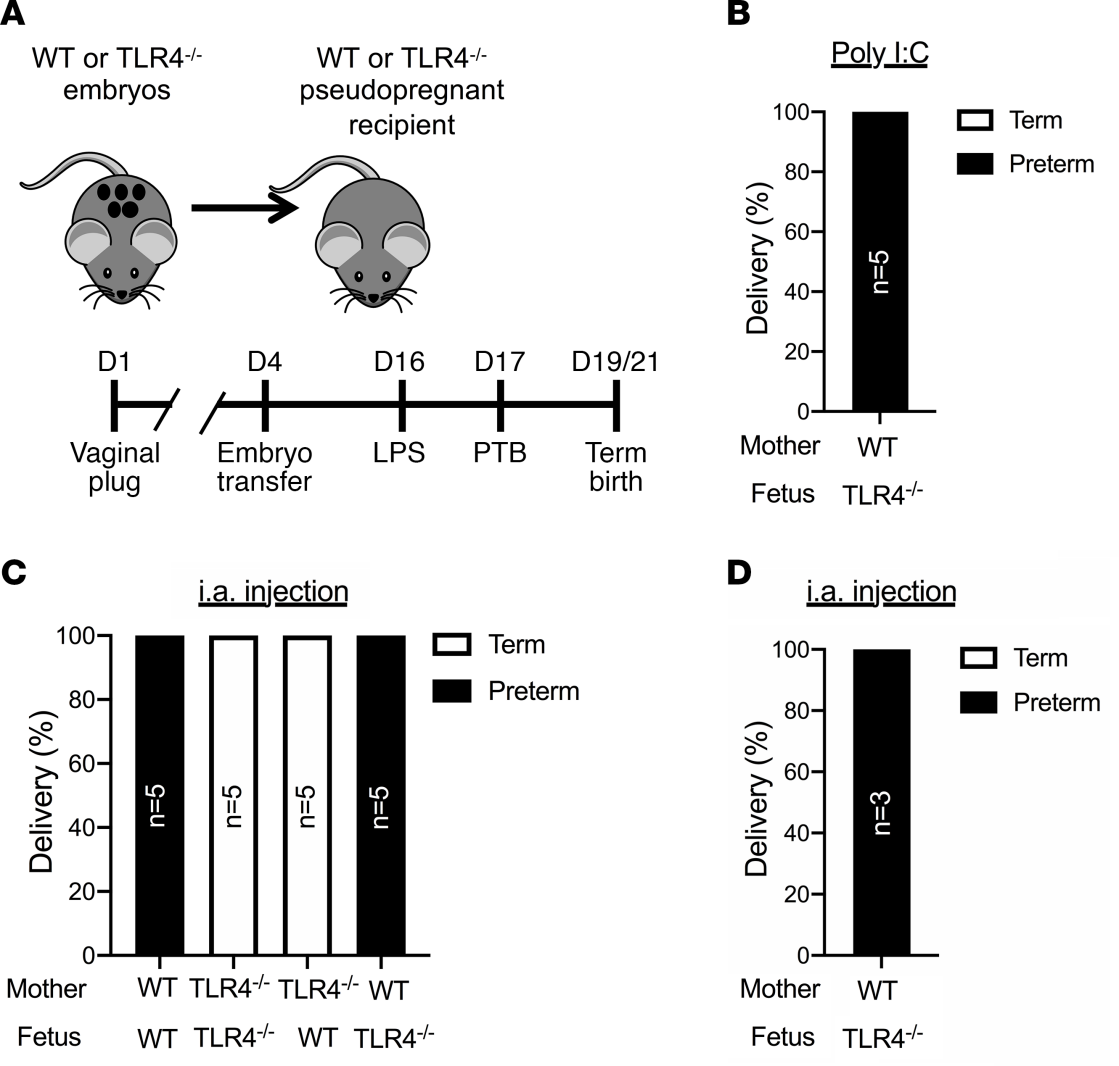
Maternal TLR4 expression is sufficient to induce preterm birth. (**A**) Schematic overview of the approach used to evaluate inflammation-induced PTB in TLR4-knockout and WT pregnancies following reciprocal embryo transfer. (**B**) Pregnancy day 16 poly(I:C) challenge in TLR4^–/–^ mothers carrying WT pups (*n* = 5); instances of PTB were quantified. (**C**) Gravid WT and TLR4^–/–^ mice (*n* = 5/condition) carrying the indicated genotype of pup following embryo transfer were challenged i.p. with 75 μg ultrapure LPS at D16 of pregnancy and instances of PTB were quantified. χ^2^ (2 × 4 matrix) *P* = 0.0002. (**D**) Gravid WT mice (*n* = 3) carrying TLR4^–/–^ pups following embryo transfer were challenged i.a. with 5 μg ultrapure LPS at day 16 of pregnancy and instances of PTB were quantified.

To formally determine the necessity of maternal TLR4 expression in induction of PTB, we next performed reciprocal embryo transfers between WT and TLR4^–/–^ mice and subsequently challenged them with TLR ligands (Figure 3A). Susceptibility of TLR4^–/–^ pregnancies to the TLR3 ligand poly(I:C) remained intact with 100% of PTB (Figure 3B). As a control for potential alterations in signaling and inflammatory responsiveness associated with embryo transfer procedure, WT or TLR4^–/–^ embryos were transferred to mothers of the same genotype. In agreement with the results of the natural mating strategy, LPS challenge of WT mothers carrying WT embryos resulted in PTB (5/5 mice), and TLR4^–/–^ mothers carrying TLR4^–/–^ embryos were protected from LPS-induced PTB (Figure 3C). When TLR4 expression was restricted to the fetus, in the context of WT embryo transfer to TLR4^–/–^ mothers, protection from PTB was observed following i.p. LPS challenge (Figure 3C), consistent with the previous outcome that TLR4^–/–^ mothers carrying heterozygous pups were protected from LPS-induced PTB (Figure 2E). Restriction of TLR4 to the maternal compartment, in the context of TLR4^–/–^ embryo transfer to WT mothers, resulted in susceptibility to LPS-driven PTB by either systemic (Figure 3C) or amniotic route of challenge (Figure 3D). These data demonstrate that maternal, and not fetal, TLR4 expression is necessary and sufficient for both i.p. and i.a. LPS-induced PTB.

### Activation of TLR4 on immune cells is sufficient to induce preterm birth.

**Figure 4:**
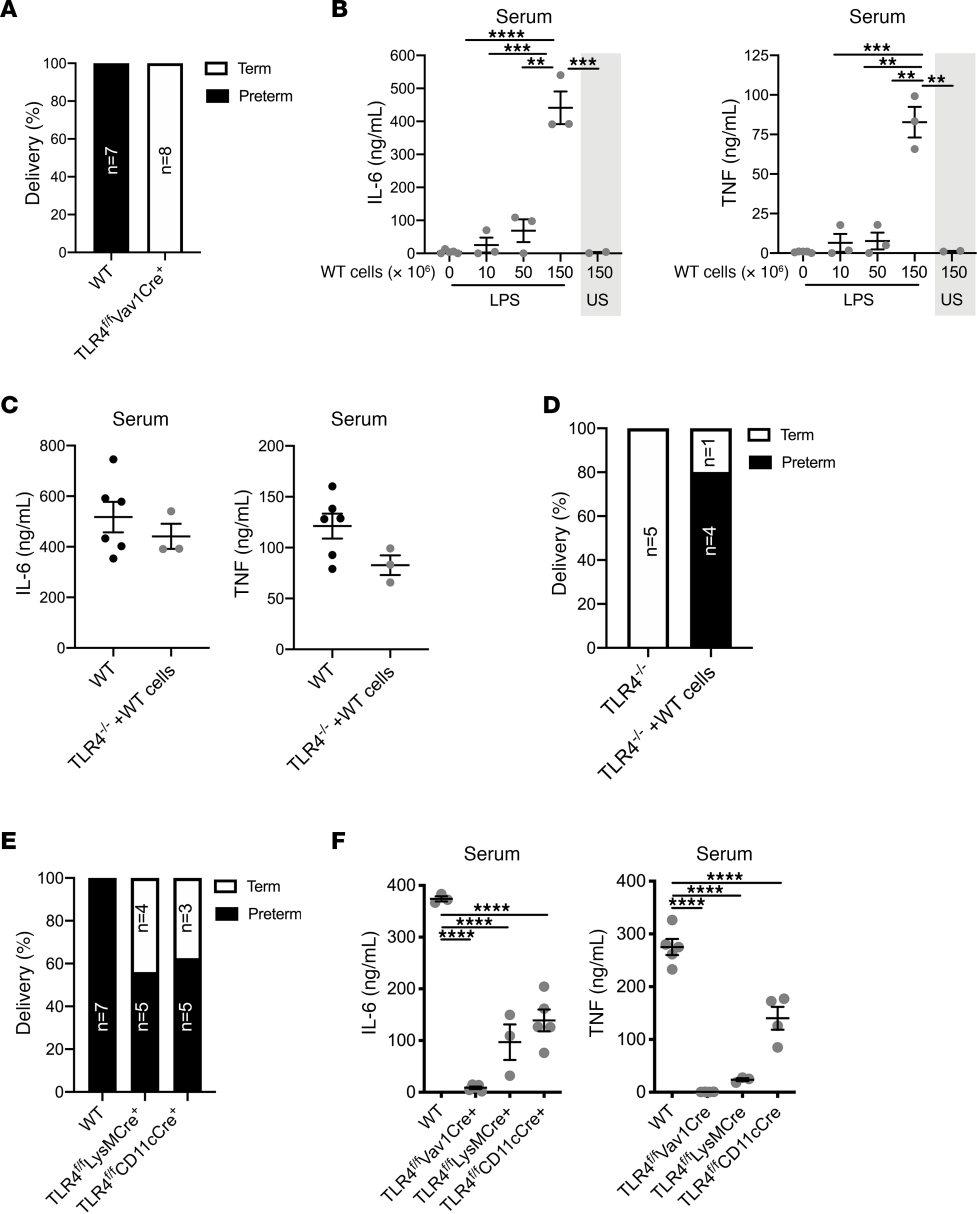
Activation of TLR4 on immune cells contributes to preterm birth. (**A**) Gravid TLR4*^fl/fl^* Vav1-Cre and WT female mice (*n* = 5–8/condition) were challenged i.p. with 75 μg ultrapure LPS at day 16 of pregnancy and instances of PTB were quantified. Fisher’s exact test *P* = 0.0002. (**B**) TLR4^–/–^ mice received WT in vitro–derived macrophages and dendritic cells by passive transfer (*n* = 2–5/condition) as indicated, and 2 hours later mice were challenged with 75 μg ultrapure LPS or saline. Serum levels of IL-6 and TNF were measured by IVCCA. Data represent average ± SEM. ANOVA **P < 0.01, ***P < 0.001, ****P < 0.0001. (**C**) TLR4^–/–^ mice received 150 × 10^6^ WT in vitro–derived macrophages and dendritic cells by passive transfer (*n* = 3), and 2 hours later mice were challenged with 75 μg ultrapure LPS alongside WT controls (*n* = 6). Serum levels of IL-6 and TNF were measured by IVCCA. (**D**) Gravid TLR4^–/–^ mice received 150 × 10^6^ WT in vitro–derived macrophages and dendritic cells by passive transfer (*n* = 5) on day 16 of pregnancy and 2 hours later were challenged with 75 μg ultrapure LPS alongside gravid TLR4^–/–^ controls (*n* = 5). Instances of PTB were quantified. Fisher’s exact test *P* = 0.0476. (**E**) Gravid TLR4*^fl/fl^* LysM-Cre, TLR4*^fl/fl^* CD11c-Cre, and WT female mice (*n* = 7–9/condition) were treated with LPS at day 16 of pregnancy and instances of PTB were quantified. χ^2^ *P* = 0.1244. (**F**) WT, TLR4*^fl/fl^* Vav1-Cre, TLR4*^fl/fl^* LysM-Cre, and TLR4*^fl/fl^* CD11c-Cre mice were treated with ultrapure LPS and serum levels of IL-6 and TNF were measured by IVCCA (*n* = 3–5/condition). Data represent average ± SEM. ANOVA of each Cre^+^ condition compared with Cre^–^ followed by Tukey’s correction. ****P < 0.001.

The results of the previous experiments established the dominance of maternal inflammatory response in induction of PTB. However, the TLR4-expressing cell type(s) necessary for susceptibility to LPS-driven PTB remained to be defined. The contributions of different cell types (e.g., trophoblasts, immune cells, decidual stromal cells) in sensing LPS and promoting inflammation required for induction of PTB have been widely debated (29, 57–60). To address the contribution of immune cell TLR4 expression to LPS-induced PTB, we employed TLR4*^fl/fl^* mice (61) bred with Vav1-Cre mice (62). The Vav1-Cre system has been reported to delete robustly in maternal hematopoietic cells with possible activity reported in endothelial cells (63–65). The functional efficiency of Cre-mediated deletion of TLR4 in immune cells was confirmed by LPS stimulation of isolated peritoneal macrophages. As expected, TLR4*^fl/fl^* Vav1-Cre^+^ cells did not produce IL-6 in response to LPS stimulation but did produce similar cytokine levels as Cre^–^ cells when stimulated with a TLR2 ligand, Pam3Cys (Supplemental Figure 3A). As shown in Figure 4A, Vav1-Cre–mediated deletion of TLR4 fully protected from LPS-driven PTB. This outcome suggested maternal immune and/or endothelial cell–associated inflammation (29) was an important contributor to LPS-driven PTB.

To define the sufficiency of immune cells’ LPS sensing in promoting inflammation and induction of PTB, we employed passive transfer of WT in vitro–derived macrophages and dendritic cells (28, 66) to gravid TLR4^–/–^ mice. Systemic levels of proinflammatory cytokines (e.g., IL-6, TNF) produced after LPS challenge were proportional to the number of WT cells transferred (Figure 4B), and transfer of 150 × 10^6^ in vitro–derived macrophages and dendritic cells to TLR4^–/–^ mice resulted in similar levels of systemic IL-6 and TNF as detected in WT mice (Figure 4C). Notably, transfer of WT macrophages and dendritic cells into pregnant TLR4^–/–^ mice was sufficient to induce PTB following LPS challenge (Figure 4D). Importantly, PTB did not occur in the absence of LPS challenge (cells alone) or in TLR4^–/–^ pregnancies where low numbers of WT macrophages and dendritic cells were transferred before LPS challenge (Supplemental Figure 4).

The ability of WT in vitro–derived macrophages and dendritic cells to induce PTB following LPS challenge raised the question as to whether a single subset of immune cells was more relevant in driving such a response. TLR4*^fl/fl^* mice were crossed with LysM-Cre (traditionally employed for deletion in macrophage/neutrophil subsets) (67) or CD11c-Cre (traditionally employed for deletion in dendritic cells) (68) mice, and cytokine production in response to LPS was tested in vitro (Supplemental Figure 3B). Deletion of TLR4 on macrophages/neutrophils or dendritic cells reduced sensitivity to LPS-induced PTB but did not provide protection for the majority of pregnancies (Figure 4E). Protection from LPS-driven PTB inversely correlated with the degree of systemic proinflammatory cytokine production induced in the setting of flox/Cre-mediated deletion of TLR4 (Figure 4F). These data suggest that both macrophages/neutrophils and dendritic cells may contribute to the TLR ligand–induced inflammatory responses that drive PTB.

## Discussion

PTB remains the leading cause of neonatal morbidity and mortality (1, 2). Infection and resulting inflammation are recognized as major underlying causes of PTB. However, a basic understanding of the critical cell types driving PTB, the role of maternal and fetal sensing of inflammatory stimuli, and how those responses may vary depending on the anatomical location of inflammation have not been well understood and represent a major gap in knowledge (9).

Previous studies have shown differences in maternal response related to route of inflammatory challenge (22, 23, 32). It has been proposed that i.a. challenge, compared with i.p. challenge, is more clinically relevant because presence of microbial products in amniotic fluid has been causally linked to PTB (22, 69). However, the cell types involved in detecting and responding to such challenge remain unclear. A complicating factor in elucidating specific cell types and molecular pathways involved in induction of PTB has been the use of impure reagents that stimulate multiple pathways. Further, the examination of these pathways at various time points postchallenge is clearly needed. Consistent with findings from other groups, we found that commonly purchased, not ultrapurified, LPS was not specific for TLR4 activation, and it robustly induced proinflammatory cytokine production through activation of other TLRs (70, 71). In addition to LPS purity, previous work has demonstrated differences in PTB response when LPS from different *E*. *coli* serotypes was used (36). Of note, the exact dose of LPS required to induce PTB could change depending on the time of gestation examined (72). In addition to bacterial infections, viral infections during pregnancy are known to drive PTB and to predispose to secondary bacterial infection, potentially through changes in TLR expression and augmentation of inflammatory responses systemically (43) or at the materno-fetal interface (73, 74). The contribution of a fetal response in this context (where multiple microbial products and pathways are involved), however, has not been examined. These studies are warranted because the contribution of fetal inflammatory sensing in such matrixed settings of PTB may be more impactful than observed in the present study.

We found that both i.p. and i.a. LPS challenge induced PTB depending on maternal expression of TLR4. Upregulation of markers indicative of macrophage infiltration/activation and uterine inflammation were detected following both routes of challenge. Proinflammatory cytokine expression regulates pregnancy and the timing of parturition (75, 76). Consistent with the role of cytokines in initiation of labor, we found increased expression of *Il6* and *Tnf* following LPS challenge in conditions that led to PTB. Proinflammatory cytokine expression can upregulate *Ptgs2* (Cox-2), which is associated with uterine contractility and labor (77). Upregulation of *Ptgs2* (Cox-2) was detected in both the i.p. and the i.a. LPS injection models, consistent with proinflammatory cytokine–driven induction of labor.

Umbilical cord plasma recovered from human cases of PTB was found to contain higher levels of IL-6 than umbilical cord plasma from term births (78). It has been proposed that a fetal proinflammatory response is not balanced by reciprocal antiinflammatory cytokine production, which has deleterious effects on the fetus (79, 80). Small amounts of LPS can reach the fetal compartment following either i.p. (20) or i.a. challenge, and fetal cells were able to respond to LPS, but detection of proinflammatory cytokines in amniotic fluid was low compared with maternal serum at the time point examined. This dampened fetal response has also been reported in other mouse models of PTB (72, 81), as well as pregnant rats following LPS challenge (79), and observed in the context of fetal inflammatory response syndrome (55, 56). The difference in inflammatory response detected in serum compared with amniotic fluid could underlie the observation that maternal, but not fetal, immune sensing was both necessary and sufficient for induction of PTB following LPS challenge.

In this report, we show that maternal expression of TLR4 on immune cells contributed to LPS-induced PTB. Specifically, the results of the embryo transfer experiments, where maternal TLR4 expression was necessary and sufficient for LPS-induced PTB, supports the dominant role of maternal, rather than fetal, immune cell sensing of inflammatory stimuli in inflammation-driven PTB. Notably, this is consistent with previous reports suggesting a role for maternal TLR4 expression in the context of pregnancy complications (33, 58–60, 82). Vav1-Cre–mediated genetic recombination is believed to delete in both hematopoietic cells and endothelial cells because these cells share a common progenitor (62, 63). Endothelial cells in the decidual bed are implicated in induction of PTB following LPS challenge (29). However, in our studies, passive transfer of WT in vitro–derived macrophages and dendritic cells to pregnant TLR4^–/–^ mice was sufficient to enable LPS-driven PTB. Additionally, our finding that LysM-Cre– and CD11c-Cre–mediated deletion of TLR4 led to reduced sensitivity to LPS-induced PTB demonstrates the contribution of immune cells in induction of inflammation-driven PTB. Considering these outcomes, it is likely that the complete protection observed in TLR4*^fl/fl^* Vav1-Cre^+^ mice may be largely attributed to a loss of inflammatory sensing in immune cells in our experimental model. Additional experiments employing heterozygous pregnancies in this context would further delineate the contribution of maternal versus fetal immune cells. Further, future studies directly analyzing TLR4 expression and function in uterine vessels of TLR4*^fl/fl^* Vav1-Cre and immune cells of TLR4*^fl/fl^* Tie2-Cre mice are clearly needed.

Experimental models that reflect both systemic maternal infections (e.g., influenza, sepsis) and intrauterine infections (83, 84) are clearly needed. However, the differences among mammalian species in terms of pregnancy and parturition are apparent (85). Placental structure, gestation period, and progesterone withdrawal before parturition all represent points of divergence across mammalian pregnancies (86). Despite limitations in drawing conclusions about human pregnancy from the mouse model, the mouse offers significant advantages as a model for inflammation-induced PTB. Pathways regulating immune responses are highly conserved between mice and humans (27). Thus, immune mechanisms that contribute to inflammation-induced PTB in the mouse are likely to be significant in human pregnancy.

Immune cells were robust producers of proinflammatory cytokines and were found to be the dominant contributors to LPS-driven PTB. Macrophages have previously been implicated as critical for induction of PTB following TLR9 activation (18). We found reduced sensitivity to LPS-induced PTB when TLR4 was deleted in macrophages/neutrophils or dendritic cells. LysM-Cre has been reported to inefficiently delete in all macrophages (67), which may contribute to the partial phenotype we observed. These results suggest that macrophages/neutrophils and dendritic cells contribute to sensing inflammatory stimuli and mounting a proinflammatory response.

In summary, in this brief report, we examined the differential role of TLR4-driven immune response in the maternal and fetal compartments. Importantly, we identified a fetal inflammatory response following LPS challenge. Our data demonstrate that the maternal response is the dominant driver of inflammation-induced PTB because maternal TLR4 expression was necessary for systemic and local LPS-driven induction of PTB. Moreover, we found that TLR4 expression on immune cells of the maternal compartment was sufficient for TLR4-driven induction of PTB. These data argue that TLR4-driven induction of PTB depends on maternal immune activation in the context of systemic and local inflammation. Further mechanistic studies focusing on specific maternal inflammatory pathways central to induction of PTB are clearly needed to formally delineate inflammatory targets central to activation of maternal responses.

## Methods

### Reagents.

All cell culture reagents were endotoxin free to the limits of detection of the Limulus amebocyte lysate assay (Lonza) at the concentrations employed. Except where indicated, all TLR ligands used in in vitro and in vivo studies [LPS; *E*. *coli* 0111:B4, Pam3Cys, Pam2Cys, and poly(I:C)] were ultrapure grade (InvivoGen). Where indicated, *E*. *coli* LPS (0111:B4 MilliporeSigma) was used.

### Cell culture.

HEK293 cells stably expressing CD14 and TLR2 (H2.14) were used and treated as described before (ATCC) (87). Briefly, H2.14 cells were cultured in complete culture medium (RPMI-1640 medium from Gibco, Thermo Fisher Scientific, supplemented with 10% FCS from Gibco, Thermo Fisher Scientific; 1% l-glutamine from MilliporeSigma; and 50 μg/mL gentamicin from Cellgro) and 5 μg/mL puromycin (Calbiochem) for selection purposes. Cells were stimulated with 10 ng/mL standard LPS (MilliporeSigma), 10 ng/mL ultrapure LPS (InvivoGen), or 1.5 μg/mL Pam2Cys (InvivoGen), or were mock-stimulated with media alone for 24 hours and cell-free supernatants were collected. IL-8 levels were quantified by ELISA (BD) according to the manufacturer’s instructions.

Murine thioglycollate elicited peritoneal macrophages (EPMs) were generated using standard protocol (43, 87). EPMs (1 × 10^6^ cells/well) were mock-stimulated or stimulated with ultrapure LPS (100 ng/mL) or Pam3Cys (1.5 μg/mL) for 18 hours, and cytokine production (IL-6) was determined by ELISA (BD).

MEFs were generated from E13 embryos. Briefly, embryos were removed from the uterus and washed in 70% ethanol and PBS. Fetal tissue was minced and tissue was digested in 0.25% trypsin/EDTA for 15 minutes. Cells were maintained in DMEM supplemented with 10% FBS, 1% l-glutamine, and 1% penicillin/streptomycin. MEFs (1 × 10^6^ cells/well) were mock-stimulated or stimulated with ultrapure LPS (100 ng/mL) or Pam3Cys (1.5 μg/mL) for 4 hours, and cytokine production (IL-6) was determined by ELISA (BD).

### Mice.

Female mice (WT, TLR4^–/–^, TLR4*^fl/fl^* Vav1-Cre, TLR4*^fl/fl^* LysM-Cre, TLR4*^fl/fl^* CD11c-Cre) on a C57BL/6J background (bred in-house), were mated with fertile male mice of the same strain (61, 88). Animals were housed in a specific pathogen–free animal facility at Cincinnati Children’s Hospital Medical Center (CCHMC) and handled in high-efficiency particulate-filtered laminar flow hoods with free access to food and water. For studies that used heterozygous pregnancies, TLR4^–/–^ mice were mated with WT mice as indicated. For all pregnancy studies the presence of a vaginal plug marked day 1 of pregnancy. Parturition events were monitored by visual inspection twice daily on days 17–21 and defined as complete delivery of pups (43, 89).

### Embryo transfers.

Blastocysts were collected from day 4 postcopulation WT or TLR4^–/–^ mice for embryo transfer. Pseudopregnant recipients were generated by mating females with vasectomized males. Blastocysts were transferred into day 4 postcopulation uteri of WT or TLR4^–/–^ pseudopregnant recipients as described previously (21).

### Passive transfer of in vitro–derived macrophages and dendritic cells.

Bone marrow cells were derived from the femurs of WT mice and cultured in complete culture medium (RPMI-1640 medium from Gibco, Thermo Fisher Scientific, supplemented with 10% FCS from Gibco, Thermo Fisher Scientific; 1% l-glutamine from MilliporeSigma; and 50 μg/mL gentamicin from Cellgro) and 10 ng/mL GM-CSF (PeproTech). Media were added on day 3. This protocol generates both macrophage and dendritic cell populations (90, 91). On day 6, cultured cells were collected. The indicated number (10 × 10^6^, 50 × 10^6^, 150 × 10^6^) of cells were transferred i.p. to the recipient mouse 2 hours before LPS challenge (92, 93). IVCCA (43, 88, 89, 94–96) was used to quantify systemic IL-6 and TNF levels. Briefly, biotinylated capture antibodies IL-6 (MP5-32C11) and TNF (TN3) (eBioscience, Thermo Fisher Scientific) were injected i.p. 3 hours before TLR ligand challenge (75 μg ultrapure LPS), and serum cytokine levels were determined 4 hours later.

### Preterm birth.

On day 16 of gestation, gravid female mice were challenged i.p. or i.a. with LPS (InvivoGen or MilliporeSigma where depicted) or saline (unstimulated) at indicated concentrations. PTB was defined as parturition within 24 hours after challenge (all pups deceased). Term birth was defined as parturition between days 19 and 21 (all pups alive) (43, 89). In all instances where PTB did not occur on day 17, live pups were born at term.

### I.a. injections.

For i.a. injections, mice were anesthetized with isoflurane (5% induction; 1.5% maintenance) in oxygen and positioned supine on the stage with abdominal hair removed and ultrasound gel applied to the abdomen. Scans and injection guidance (2 amniotic sacs per pregnancy; 1 left horn and 1 right horn) were performed with the Vevo 2100 ultrasound scanner (FUJIFILM VisualSonics) and MicroScan transducers. Fetal viability (temperature and heart rate) and injection success were determined following injection of 100 μL saline into the amniotic cavity. Injection success was visualized by MicroMarker contrast agent (FUJIFILM VisualSonics). All studies were done in collaboration with professional sonographers from the CCHMC Cardiovascular Imaging Core Research Laboratory.

### Cytokine quantification.

In vivo, systemic IL-6 and TNF levels were quantified using IVCCA (43, 88, 89, 94–96). Briefly, biotinylated capture antibodies IL-6 (MP5-32C11) and TNF (TN3) (eBioscience, Thermo Fisher Scientific) were injected i.p. 3 hours before TLR ligand challenge (25 μg LPS), and serum cytokine levels were determined 4 hours later.

For comparison of maternal serum and amniotic fluid levels of IL-6, samples were collected 6 hours following i.p. (75 μg LPS) or i.a. (5 μg LPS total, administered as 2 × 2.5 μg injections) challenge. Amniotic fluid of every sac for 1 uterine horn was pooled (2 samples per mouse, right and left horns), and IL-6 levels were quantified by ELISA (BD).

### Gene expression.

Uterine samples consisting of maternal decidua and myometrium were collected 6 hours or 12 hours after ultrapure LPS challenge on day 16 of gestation. For quantification of mRNA expression in murine samples, cells/tissues were homogenized in TRIzol (Invitrogen, Thermo Fisher Scientific), RNA was extracted, and cDNA was generated and quantified as previously described (89, 94) using Light Cycler 480 II (Roche Diagnostics). The following murine primers were obtained from National Center for Biotechnology Information Harvard PrimerBank (https://pga.mgh.harvard.edu/primerbank/) and used for our experiments: *Cd68*: AATGATGAGAGGCAGCAAGAGG and CTTCCCACAGGCAGCACAG; *Ccl2*: TGTCTGGACCCATTCCTTCTTG and AGATGCAGTTAACGCCCCAC; *Ccl4*: TCTCTCCTCTTGCTCGTGGC and GAATACCACAGCTGGCTTGGA; *Ptgs2* (Cox-2): AATGACCTGATATTTCAATTTTCCATC and ACTGGGCCATGGAGTGGAC; *Il6*: TGGTACTCCAGAAGACCAGAGG and AACGATGATGCACTTGCAGA; *Tnf*: CCAGACCCTCACACTCAGATCA and CACTTGGTGGTTTGCTACGAC; *Ifnb*: TCCAGCTCCAAGAAAGGACG and TTGAAGTCCGCCCTGTAGGT; *Isg15*: GTCACGGACACCAGGAAATC and AAGCAGCCAGAAGCAGACTC; *Irf7*: AGCATTGCTGAGGCTCACTT and TGATCCGCATAAGGTGTACG; *Il1b*: TGTGCTCTGCTTGTGAGGTGCTG and CCCTGCAGCTGGAGAGTGTGGA; and *Bactin*: GGCCCAGAGCAAGAGAGGTA and GGTTGGCCTTAGGTTTCAGG. Single product quantitative PCR was validated by melt curve analysis. Each reaction contained 2 μL of cDNA (25 ng/μL) and 8 μL of master mix consisting of SYBR Green PCR Master Mix (Life Technologies, Thermo Fisher Scientific), 0.5 μM 5′ and 3′ primers, and ribonuclease-free water. Data were normalized to β-actin mRNA expression and expressed as ΔΔCt using the formula mRNA level = 1.8^(Ctβ-actin^** ^–^^Cttarget^** ^gene)^ × 100,000.

### Histology of reproductive tissues.

Samples consisting of connected myometrium, decidua, placenta, and fetal membranes were collected at each implantation site from i.p. and i.a. ultrapure LPS–injected mice at 6 hours postinjection and directly placed in 10% formalin. Hematoxylin and eosin staining was performed on 5 μm sections from paraffin-embedded tissue blocks for conventional light microscopy analysis.

### Statistics.

Any data outliers were detected by GROUT in Prism 8 (GraphPad Software, Inc.) and removed from data sets. For normally distributed data, results were analyzed by 2-tailed Student’s *t* test or 1-way ANOVA followed by Tukey’s correction in Prism 8 (GraphPad Software, Inc.) as appropriate and indicated in the text. For categorical data, results were analyzed by Fisher’s exact test or χ^2^ in Prism 8 (GraphPad Software, Inc.) as appropriate and indicated in the text. All groups were analyzed at the same time as indicated by the matrix in the text. A *P* value of less than 0.05 was considered significant. All values are represented as mean ± SEM or as percentage of term or PTB induction.

### Study approval.

All studies were performed in accordance with the procedures outlined in the *Guide for the Care and Use of Laboratory Animals* (National Academies Press, 2011) and approved by the CCHMC Institutional Animal Care and Use Committee.

## Author contributions

MC, JRD, TES, MJL, VS, BW, VK, XS, and SD participated in study discussion and data generation. MC, JRD, TT, and SD participated in analysis and interpretation of data. MC, JRD, and SD participated in the conception and design of the study and wrote the manuscript. SD obtained the funding. All authors have reviewed the manuscript and approve the final version.

## Supplementary Material

supplemental data

## Version 1 11/19/2020

Electronic publication
