# Supplementary material for: Maternal regulation of inflammatory cues is required for induction of preterm birth
Source: JCI Insight. 2020 Nov 19;5(22):e138812. doi: 10.1172/jci.insight.138812 (PMC7710297; doi:10.1172/jci.insight.138812)
Supplement: supplemental data [file jciinsight-5-138812-s037.pdf]

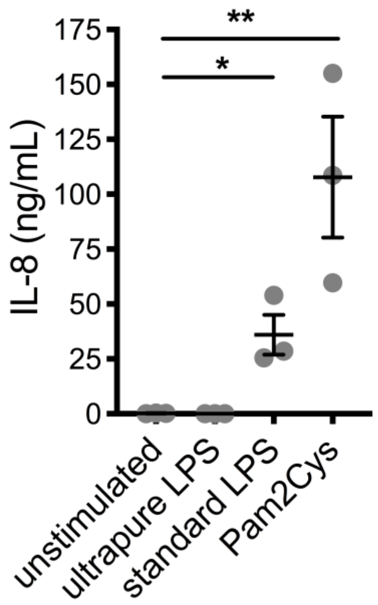

Supplementary Figure 1. LPS purity impacts specificity and amount of inflammatory cytokine production.

H2.14 cells (n = 3/condition) were stimulated with standard (non-ultrapurified) LPS (10 ng/mL), ultrapure LPS (10 ng/mL), or Pam2Cys (1.5 μg/ml) for 24 hours and the amount of IL-8 produced was quantified by ELISA (n = 3/condition). Data represent average value ± SEM. ANOVA followed by Tukey's correction. *P<0.05, **P<0.01.

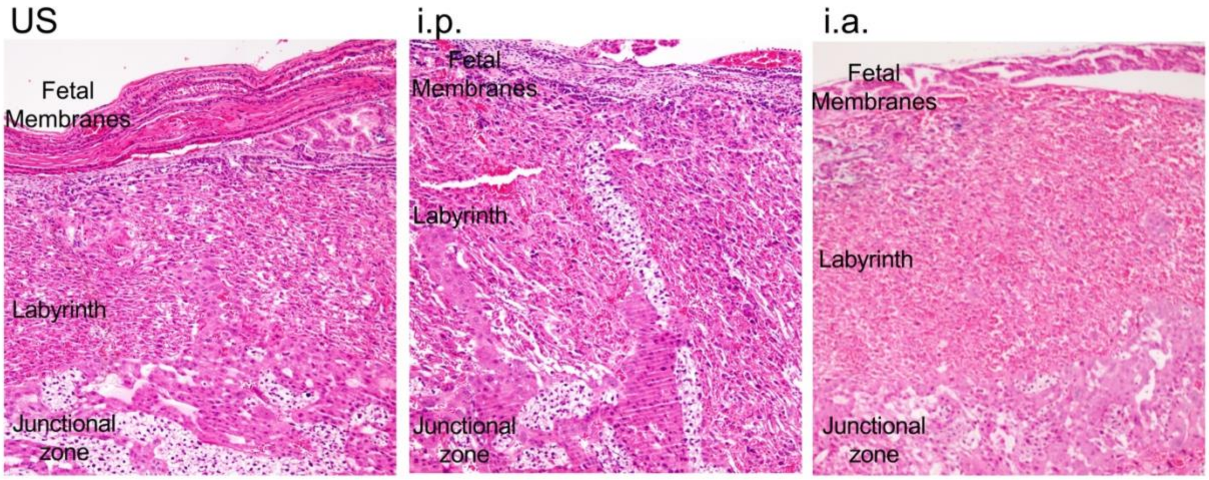

Supplementary Figure 2. Representative placentas after LPS challenge. Gravid WT mice were challenged with saline (US = unstimulated) or ultrapure LPS by i.p or i.a. injection for 6 h and placentas (n = 9/condition) were processed for histological examination (H&E staining, 10x). A representative staining for each condition is depicted.

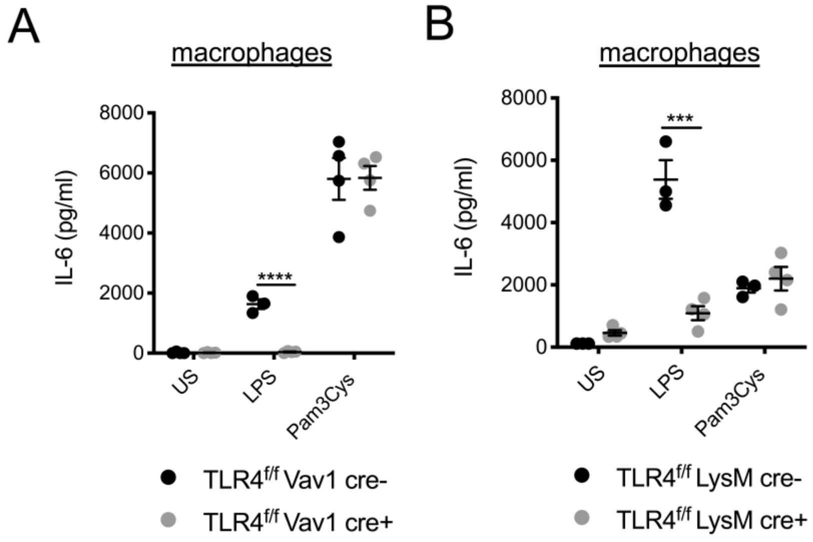

Supplementary Figure 3. Vav1Cre and LysMCre mediated deletion of TLR4 reduces inflammatory response to LPS.

(A) WT and TLR4$^{f/l}$Vav1Cre murine peritoneal macrophages (n=3-4/genotype), were unstimulated (US) or treated with ultrapure LPS (100 ng/mL) or Pam3Cys (1.5 μg/ml) and IL-6 levels were quantified by ELISA. Data represent average value ± SEM. **(B)** WT and TLR4$^{f/f}$LysM-Cre murine peritoneal macrophages (n=3/genotype), were treated with ultrapure LPS (100 ng/mL) or Pam3Cys (1.5 μg/ml) and IL-6 levels were quantified by ELISA. Data represent average value ± SEM. (**A-B**) Student's t-test of each Cre^{+} compared to Cre^{-} treatment group. ***P<0.001, ****P<0.0001.

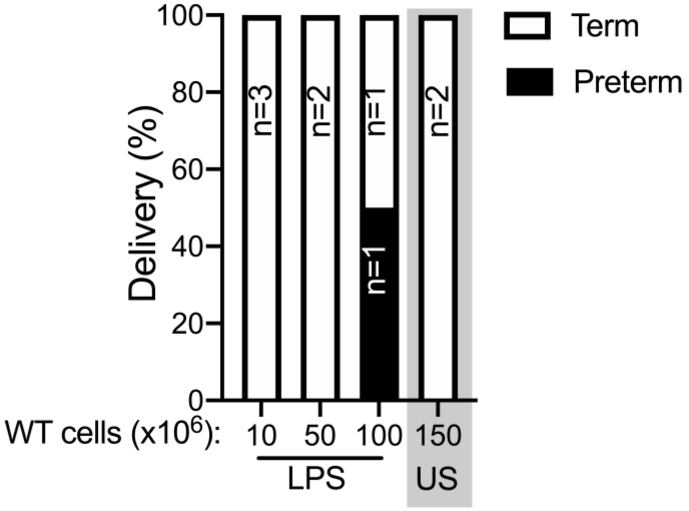

Supplementary Figure 4. Passive transfer of WT cells to gravid TLR4$^{-/-}$ mice below the threshold required for preterm birth induction.

Gravid TLR4$^{-/-}$ mice received WT in vitro-derived macrophages and dendritic cells by passive transfer (n = 2-3/condition) on D16 of pregnancy and 2 h later were challenged with 75 µg ultrapure LPS or saline (US = unstimulated). Instances of PTB birth were quantified. Chi-square (2x4 matrix) p=0.2683.
